# Comparison of Protein Phosphatase Inhibition Assay with LC-MS/MS for Diagnosis of Microcystin Toxicosis in Veterinary Cases

**DOI:** 10.3390/md14030054

**Published:** 2016-03-09

**Authors:** Caroline E. Moore, Jeanette Juan, Yanping Lin, Cynthia L. Gaskill, Birgit Puschner

**Affiliations:** 1 cemoore@ucdavis.edujjuan8042@gmail.comyaplin@ucdavis.edu; 2 cynthia.gaskill@uky.edu

**Keywords:** blue-green algae, cyanobacteria, diagnosis, microcystins, protein phosphatase inhibition assay, veterinary toxicology

## Abstract

Microcystins are acute hepatotoxins of increasing global concern in drinking and recreational waters and are a major health risk to humans and animals. Produced by cyanobacteria, microcystins inhibit serine/threonine protein phosphatase 1 (PP1). A cost-effective PP1 assay using *p*-nitrophenyl phosphate was developed to quickly assess water and rumen content samples. Significant inhibition was determined via a linear model, which compared increasing volumes of sample to the log-transformed ratio of the exposed rate over the control rate of PP1 activity. To test the usefulness of this model in diagnostic case investigations, samples from two veterinary cases were tested. In August 2013 fifteen cattle died around two ponds in Kentucky. While one pond and three tested rumen contents had significant PP1 inhibition and detectable levels of microcystin-LR, the other pond did not. In August 2013, a dog became fatally ill after swimming in Clear Lake, California. Lake water samples collected one and four weeks after the dog presented with clinical signs inhibited PP1 activity. Subsequent analysis using liquid chromatography-mass spectrometry (LC-MS/MS) detected microcystin congeners -LR, -LA, -RR and -LF but not -YR. These diagnostic investigations illustrate the advantages of using functional assays in combination with LC-MS/MS.

## 1. Introduction

Large blooms of cyanobacteria (also known as blue-green algae) can release a variety of toxic metabolites, known as cyanotoxins [1]. Blooms are triggered by warm temperatures and high levels of nitrogen and phosphorus from urban effluent and agricultural run-off [1]. The acute hepatotoxic microcystins (MCs) are well known cyanotoxins. MC-LR (named for leucine and arginine at the two variable amino acids in this cyclic heptapeptide) was the first identified MC to be associated with toxicity and is therefore the most tested for and studied MC. Since then over 90 MC variants have been identified [2]. Acute MC exposure can result in dermatitis, gastrointestinal upset and liver failure and even death after high dose exposures [3]. Currently there are no legally enforceable US federal regulations for cyanobacteria or cyanotoxins in drinking water, recreational waters, or in cyanobacteria supplement products.

In response to continued increases in harmful cyanobacterial blooms in the United States the US Environmental Protection Agency (USEPA) added cyanobacteria and cyanotoxins to the Candidate Contaminant List of unregulated contaminants [4]. In addition, the USEPA issued Health Advisories for MC-LR in drinking water in June 2015 [4]. The World Health Organization has a recommended maximum level of 1 µg/L of MC-LR (free and cell bound) in drinking water for humans [5], which was based on the results of an acute hepatotoxicity with MC-LR in mice [6]. This provisional guideline does not take into account species differences, more hydrophobic congeners, chronic exposure risk, or target organs other than the liver. In veterinary medicine there is a particular concern for livestock [3,7,8,9], canine [10,11,12,13,14,15] and wildlife exposure to cyanotoxins via surface drinking water from contaminated ponds, lakes and rivers. As these species are typically exposed to environmental contaminants before humans, they may act as sentinels of human health risk. The availability of rapid and low cost assays is therefore essential to evaluate the risk of contaminated drinking water to livestock and companion animals and to guide diagnostic investigations of cyanotoxin exposures.

To confirm MC toxicosis in animals, clinical signs and/or post mortem lesions must be compatible with the analytical confirmation of MCs in biological samples. In tissues, MCs covalently bind serine/threonine protein phosphatases (PP) 1 and 2A [16,17,18]; therefore extraction of free and bound MCs from biological samples for high performance liquid chromatography (HPLC) or enzyme linked immunosorbent assays (ELISA) is costly, challenging, and may lead to false negative findings or underestimation of the true MC concentrations. As a result, most diagnostic testing relies heavily on identifying the presence of MCs in water samples. When using only HPLC or ELISA techniques to detect specific MC variant concentrations, the toxic potency of an environmental sample based on all congeners present cannot be directly determined for two key reasons: (1) with MC cross-reactivity, ELISAs may detect high concentrations of non-toxic MCs while having potentially low specificity for highly toxic variants [19] and (2) analytical techniques relying on liquid chromatography-mass spectrometry (LC-MS/MS) are limited by the availability of certified reference standards. Currently only a small number of the over 90 MC congeners are available to be included in LC-MS/MS screening assays. However, compared to ELISAs, LC-MS/MS has the benefit of being able to quantify a particular MC congener. The aforementioned diagnostic tools are not capable of determining if the detected MCs are functional (able to inhibit PPs). Therefore, to minimize the limitations of LC-MS/MS and ELISA techniques and to fully assess the toxic potential of all congeners in a sample, biological activity assays should be used in combination with LC-MS/MS and/or ELISA [20].

Protein phosphatase inhibition assays (PPIAs) were originally developed to study MC’s ability to bind PP1 and 2A [21,22]. They are ideal biological toxicity assays for environmental samples. PPIAs have become a standard method for MC detection with the use of the colorimetric substrate *p*-nitrophenyl phosphate (*p*NPP, no color) and purified PP1 [19,23]. The assay measures the rate of formation of *p*-nitrophenol (*p*NP, yellow color) by PPs over time and takes into account all PP inhibitors present in a sample. The colorimetric PPIA has been optimized for MC detection in cyanobacteria extracts using 96-well plates, and has shown acceptable correlation with HPLC data [20,24,25]. With commercially available *p*NPP and purified PP1, which is significantly cheaper than PP2A, the assay is very attractive to quickly and effectively screen for the presence of PP1 inhibitors. Currently, PPIAs are not common in veterinary diagnostic investigations. However, they have the potential to detect the presence of toxins that may escape detection with LC-MS/MS or ELISA. Therefore, the objectives of this study were (1) to standardize the PPIA using *p*NPP and purified PP1 after exposure to MC-LR and MC-LF, as hydrophilic and hydrophobic MC variant representatives, (2) to use tautomycin and okadaic acid to validate the PPIA for toxins with varying PP1 inhibitory constants (K*_i_*) (PP1 K*_i_* = 0.04, 0.33 and 118 µg/L for MC-LR, okadaic acid and tautomycin, respectively), (3) to apply a new statistical approach that takes into account the non-normal distribution of error of PPIA data in order to further establish the PPIA as a robust and simple tool and (4) to apply the PPIA assay to diagnostic investigations of canine and bovine microcystin toxicoses and compare the data to LC-MS/MS data (analytical reference standards included were MC-LR, MC-LF, MC-RR, MC-YR and MC-LA).

## 2. Results and Discussion

### 2.1. Statistical Evaluation of the PPIA with Environmental Samples and Presentation of Data

Typically, PPIA data are presented as the percent PP activity (*y*-axis) after exposure to a range of toxin concentrations (*x*-axis). In this study, however, the PPIA results are analyzed and the data are presented differently. In an effort to determine whether minimal sample preparation of environmental samples would provide a useful approach, we began by only sonicating and centrifuging samples to initiate cell lysis and remove cellular debris. This is reflected by the *x*-axis, which represents the volume of the centrifuged environmental sample analyzed. The use of this approach is based on the assumption that a greater volume of environmental sample added to the assay equates to a higher amount of toxin available to inhibit PP1. Final concentrations of PP1 and *p*NPP and total volume in a given well remained constant. We assessed four different volumes of environmental samples. The relative change in PP1 activity (*y*-axis) was determined using a linear regression model with transformed data to account for the non-normal distribution of error of our data. Traditional statistical methods used to analyze the change in PP activity as a percent of control rely on a one-way analysis of variance with a post-hoc test to determine if individual samples significantly decreased PP activity. This approach was not appropriate for our analysis due to (1) the non-normal distribution of our data and (2) the requirement to compare PP activities of environmental samples to each other for the one-way analysis of variance with a *post hoc* test; our goal was to determine whether a trend could be identified across all volumes of environmental sample tested. To accomplish this, the rate of PP1 activity (mM *p*NP produced/ng PP1/minute) after exposure to a certain volume of environmental sample was compared to the control PP1 activity (without sample exposure). By assigning the response variable as a relative PP1 activity rate, expressed as log (exposed PP1 activity rate/control PP1 activity rate), we were able to analyze a percent change in a continuous measurement (PP1 activity) with normal error without requiring further transformation. By using this new approach to transform PPIA data that takes into account the non-normal distribution of error, an appropriate application of the linear regression model can now be used to quickly determine whether the slope of the model is significant. In summary, data are presented as the relative change in PP1 activity (*y*-axis) to increasing volumes of environmental sample (*x*-axis), resulting in a statistically sound qualitative assessment of the presence of toxic PP1 inhibitors in diagnostic investigation samples.

### 2.2. Optimization of the PPIA

To optimize the PPIA the same approach and statistical method discussed above were used with four known concentrations of MC-LR, MC-LF, tautomycin and okadaic acid. Initial buffer conditions were based on previously published PPIAs [26,27] and a precipitation was formed after ten minutes, reducing assay function. This effect has been observed before [28] and it was determined that a high concentration of dithiothreitol (DTT) was the main cause for precipitation in our study as well as in the previously published paper. By reducing chemical concentrations to those listed in “Experimental Section 3.4”, buffers remained clear for the duration of the experiment (90 min). To determine the optimal PPIA conditions using PP1 and *p*NPP, a range of 0.001–1 U/mL PP1 and 1–500 mM *p*NPP was tested.

As previous PPIA work has shown [27], higher concentrations of PP1 and substrate result in a steeper activity slope over time, likely because high concentrations of enzyme and substrate may produce saturating amounts of detectable product and mask PP1 inhibition caused by low toxin concentrations. Our goal was to use a linear model to determine whether samples contained PP1 inhibitors at moderate to low concentrations. We used 0.5 U/mL PP1 and 100 mM *p*NPP final concentrations, resulting in 20%–80% inhibition of PP1 activity with 0.125–1 µg/L MC-LR and MC-LF, 1.6–13 µg/L tautomycin and 100–500 µg/L okadaic acid. All samples had a coefficient of variation (standard deviation/mean in percent) below 15%, as is desirable for PPIAs [27].

**Figure 1:**
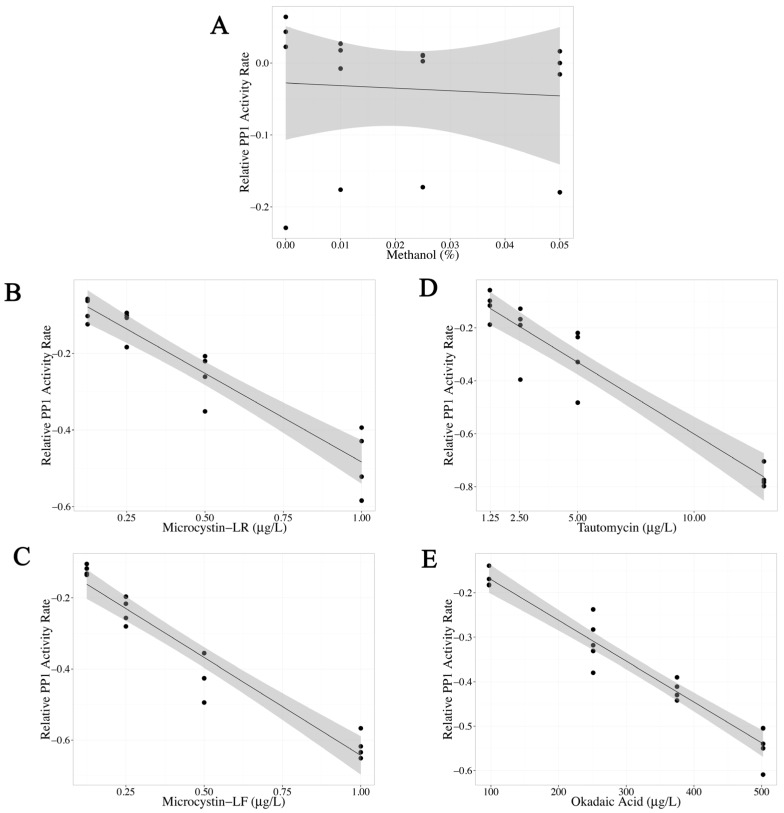
Standardization of the protein phosphatase inhibition assay (PPIA) using protein phosphatase 1 (PP1) and *p*-nitrophenyl phosphate exposed to (**A**) methanol (control); (**B**) microcystin-LR (MC-LR); (**C**) microcystin-LF (MC-LF); (**D**) tautomycin and (**E**) okadaic acid. Each PPIA (*N* = 4), ran in triplicate with a substrate blank, is represented as one point. Relative PP1 activity rate (*y*-axis), measured as log (exposed PP1 activity rate/control PP1 activity rate), was analyzed as a linear regression model (black line) with a 95% confidence interval (shaded gray area). Methanol did not significantly alter PP1 activity, while MC-LR, MC-LF, tautomycin and okadaic acid decreased PP1 activity.

PP1 activity after exposure to methanol (0%–0.05% final concentration) was also tested because all toxin standards were dissolved in 2.5% methanol before dilution. No significant change in PP1 activity was observed after methanol exposure (*p* value = 0.785, Table 1, Figure 1A). The highest methanol concentration used during assay optimization was 0.0125%, well below the highest concentration tested. Increasing concentrations of MC-LR, MC-LF, tautomycin and okadaic acid significantly inhibited PP1 activity, which is illustrated by significant negative slopes of the linear regression models (*p* value <0.0001 for all four toxins, Table 1). By comparing the magnitude of the linear regression slopes and the linear range, MC-LR and MC-LF had similar potency, followed by tautomycin (about ten times less potent than MC-LR) and okadaic acid (about 550 times less potent than MC-LR) (Table 1, Figure 1B–E).

The optimized PPIA described here was able to differentiate between MC-LR, okadaic acid and tautomycin based on a correlation between observed LRS and published PP1 K_i_ using purified rabbit PP1 and *p*NPP [29]. These data confirm that the optimized PPIA responded appropriately to pure toxins with different potencies for PP1 inhibition. Ranking the potencies from most potent to least potent based on increasing concentrations needed to inhibit 20%–80% PP1 activity is as follows: MC-LF > MC-LR > tautomycin > okadaic acid. Methanol did not significantly alter PP1 activity and was therefore not included in this ranking. These data confirm the usefulness of the PPIA to detect a variety of PP inhibitors that can be present in water samples. As okadaic acid is produced by marine dinoflagellates [30] and tautomycin is produced by the bacterium *Streptomyces spiroverticillatus* found in shellfish [31], it is unlikely either of these marine toxins would be in fresh water or surface drinking water. In addition, it is important to reiterate that the PPIA with PP1 is capable of detecting any compound that inhibits PP1, including those that may escape detection by current LC-MS/MS and/or ELISA methods. Amongst potential freshwater cyanotoxins, the only other known toxin that can inhibit PP1 is nodularin, a toxin primarily found outside of the US and in brackish water. There is concern for nodularin to be present in water suitable for drinking in Australia [32,33].

We tested two MCs, MC-LR and MC-LF, as there is little known about the inhibition potency of MC variants with different physicochemical properties. MC-LR and MC-LF were chosen based on their estimated [34] and experimental [20] octanol-water partition coefficient, lethal concentration [20], and recombinant PP1 IC_50_ [35]. In our study MC-LR and MC-LF inhibited PP1 with similar potency suggesting that the difference in hydrophobicity did not influence the toxicodynamics in the PPIA. This is in agreement with several published studies demonstrating that MC-LR and MC-LF have similar IC_50_ values for PP1 (1.2 and 1.8 µg/L [36], 1 and 3µg/L [37], 1.4 and 2.2 µg/L [38], for MC-LR and MC-LF, respectively). Recently a study using PP1 and *p*NPP to establish inhibition equivalency factors for different MC congeners in relation to MC-LR determined an IC_50_ of 2.1 µg/L for MC-LR and an IC_50_ of 359.3 µg/L for MC-LF [35]. The overall trend of lowest to highest IC_50_ was as follows: MC-LR > MC-RR > MC-YR > MC-LY > MC-LW > MC-LF. The discrepancy in this observed trend in IC_50_s for different MCs and the previously mentioned studies that showed similar IC_50_s may be a result of the source and nature of the enzyme, or the concentrations and purity of the MC variant standards, some of which were not certified in the study. Thus, it is critical that each laboratory establishes and validates assay conditions to accurately interpret results.

For the validation of the PPIA and the subsequent statistical analyses we chose two different congeners to represent the hydrophilic and hydrophobic characteristics of MCs. Once validated, one of the major objectives for the optimized PPIA was to determine overall biological toxicity in minimally processed environmental samples. To examine the PPIA toxicity data from two case studies further, LC-MS/MS data were generated and compared to PPIA data. Available standards for LC-MS/MS analysis included MC-LA (1.3/4.2 µg/L limit of detection (LOD)/limit of quantitation (LOQ)), MC-LR (0.2/0.5 µg/L LOD/LOQ), MC-LF (1.9/6.4 µg/L LOD/LOQ), MC-RR (0.2/0.6 µg/L LOD/LOQ), and MC-YR (0.3/0.7 µg/L LOD/LOQ).

### 2.3. Case A: Kentucky Case

**Figure 2:**
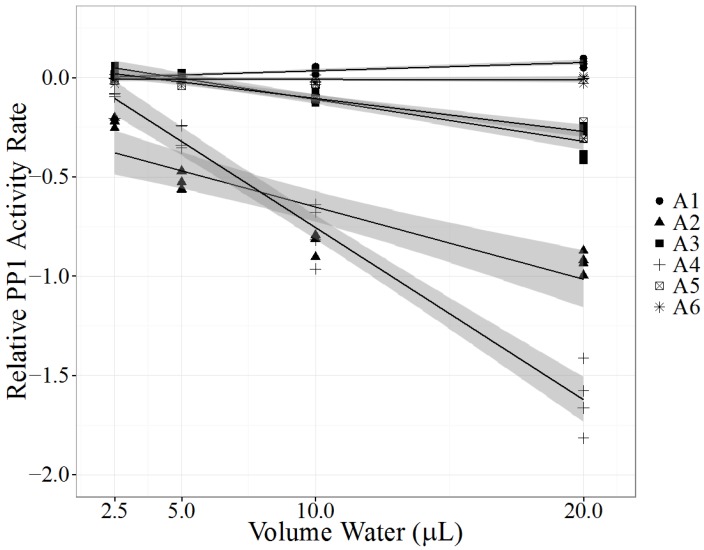
Protein phosphatase inhibition assay (PPIA) of two pond water samples (samples A1 and A2) and of rumen contents of three deceased cows (samples A3–A5) that had access to those ponds. Control rumen contents (sample A6) were collected from a calf not exposed to contaminated water. Each PPIA (*N* = 4), ran in triplicate with a substrate blank, is represented as one point. Relative PP1 activity rate (*y*-axis), measured as log (exposed PP1 activity rate/control PP1 activity rate), was analyzed as a linear regression model (black line) with a 95% confidence interval (shaded gray area). *X*-axis represents the volume of processed environmental sample added to the PPIA.

In August 2013 fifteen cattle died suddenly in or around two ponds on a farm in Nelson County, KY, USA. Pond water from two ponds (A1 and A2) and rumen contents from three cattle (A3–A5) were collected. As part of a comprehensive diagnostic investigation of a high mortality event, pond water A1, considered the likely source of cyanotoxins, was quickly analyzed by LC-MS/MS for MCs but found to be negative. A2 was initially not screened by LC-MS/MS. Based on initial diagnostic findings, a cause of death could not be determined. Fortunately with the availability of the PPIA all pond and rumen content samples were quickly analyzed. PPIA LRS results of all three rumen contents and pond A2 confirmed the presence of PP1-inhibiting compounds. Based on the PPIA results, pond A2 water and all three exposed cow rumen contents (A3–A5) were subsequently tested by LC-MS/MS. Pond water sample A1, which had no detectable MCs, significantly enhanced PP1 activity, while pond water sample A2, which contained 5.7 µg/L MC-LR, had significant PP1 inhibition (*p* < 0.0001 for A1 and A2, Table 2, Figure 2). Interestingly, the significant positive slope found with pond water A1 highlights the possibility that other compounds present in water may be capable of catalyzing the production of *p*NP by PP1. All tested rumen content samples (A3–A5) demonstrated significant PP1 inhibition (*p* < 0.0001, Table 2, Figure 2) and had detectable levels of MC-LR when analyzed by LC-MS/MS (Table 2). A control rumen content sample, collected from a ruminating calf without exposure to cyanotoxins, did not alter PP1 activity significantly (*p* = 0.851, sample A6, Table 2) and had no detectable MCs. In addition, water collected from pond A2 was examined by a phycologist (Green Water Laboratories, Palatka, FL, USA) for the presence of cyanobacteria and was found to contain three potentially toxic cyanobacteria strains: *Microcystis wesenbergii*, *Microcystis aeruginosa* and *Anabaena cf. crassa*. Both *Microcystis* spp. and *Anabaena* spp. are capable of producing microcystins. Pathological evaluation of the three necropsied cattle showed lesions consistent with acute hepatotoxicity, supporting the diagnosis of MC poisoning.

This case investigation confirmed the usefulness of the PPIA with the linear regression model to detect PP inhibitors in water and rumen content samples. The magnitude of negative LRS for each rumen content sample correlated with the MC-LR level in each sample, suggesting that MC-LR was the predominant toxic MC congener in pond A2. Direct comparison of the LRS between sample types is not possible because pond water and rumen content samples are different matrices. To the best of our knowledge, this is the first documented report of detecting PP1 inhibitors in rumen content samples using the PPIA. The PPIA offered a number of advantages over LC-MS/MS analysis. First, it was a rapid assay to obtain qualitative data about the presence of PP1 inhibitors. Second, with the use of the LRS model no standard curves were required for each PPIA. Usually, MC-LR is used as a standard to determine PP1 inhibitor concentration in terms of MC equivalents. While it is possible to use up to ten standard concentrations to estimate the amount of PP1 inhibitor present in a sample [27,35], quantitation is not necessary for these investigations and can be attempted with LC-MS/MS as a follow-up. While canine deaths due to cyanotoxin exposure are more likely to be reported, cattle may be at greater risk of cyanotoxin exposure due to their reliance on fresh water drinking ponds. In the US, 24 of a herd of 175 Hereford X Angus heifers [39] and nine of a herd of 60 Holsteins [40] died after cyanotoxin exposure. Clinical signs included nervousness, recumbency, weakness, anorexia, dehydration, ruminal atony and hypersensitivity to noise [39,40]. While there are few recent publications reporting cattle deaths from cyanotoxin exposure in the US, there have been several reports on public forums: 24 cattle died over three days in Burlington Colorado in July 1997 [39], four cows died due to cyanobacteria exposure from a pond near a ranch in Gwinnett County, Georgia in June 2012 [41] and cattle deaths were attributed to cyanotoxins in Kansas in June 2012 [42]. These reports emphasize the susceptibility of cattle to cyanotoxins, the economic impact and the need to carefully monitor water quality on ranches. Possible effects of sublethal cyanotoxin exposure on cow performance and milk and meat quality are poorly understood. However, due to the covalent binding of MCs to their targets, it is assumed that bound MCs are not biologically active. More data on the toxicokinetics of different MC congeners in cattle are needed to understand possible tissue and milk residues and the risk to consumers.

### 2.4. Case B: Clear Lake, California

**Figure 3:**
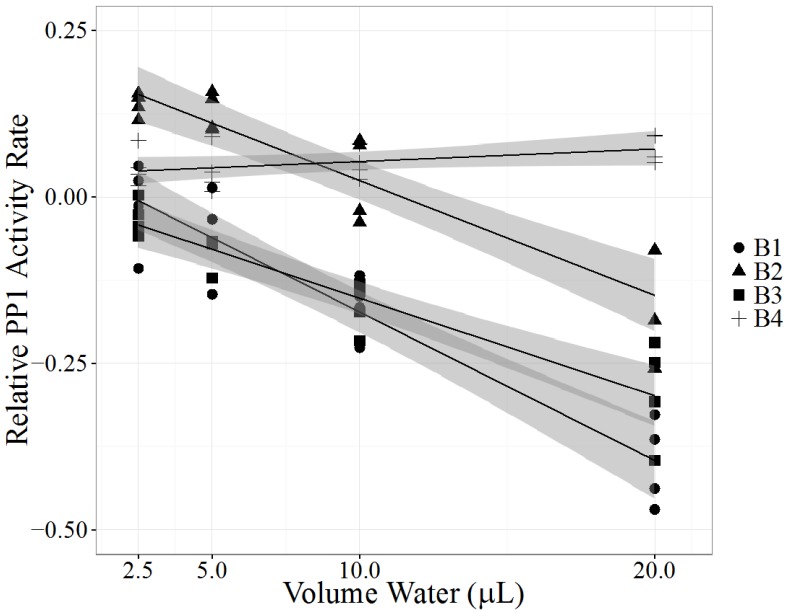
Protein phosphatase inhibition assay (PPIA) of water samples collected from Clear Lake, California in response to a dog’s death after swimming in the lake. One week after the dog swam in the lake sample B1 was collected near shore, close to the location where the dog swam. Samples B2 and B3 were collected three weeks after the first sampling; sample B2 from near shore and sample B3 200 feet from shore. Sample B4 was collected near shore four weeks after the first sampling. Each PPIA (*N* = 4), ran in triplicate with a substrate blank, is represented as one point. Relative PP1 activity rate (*y*-axis), measured as log (exposed PP1 activity rate/control PP1 activity rate), was analyzed as a linear regression model (black line) with a 95% confidence interval (shaded gray area). *X*-axis represents the volume of processed environmental sample added to the PPIA.

A Labrador retriever developed lethargy, vomiting, diarrhea and inappetence after two days of swimming in Clear Lake, CA, USA in August 2013. The dog was euthanized four days later due to severe disseminated intravascular coagulopathy (DIC). One week after the dog swam in the lake, Lake County Department of Water Resources collected and submitted water samples from near shore where the dog had accessed the lake (sample B1). Visually, the water samples contained algae. Sample B1 had detectable levels of MC-LA and MC-LR by LC-MS/MS analysis and significant potent PP1 inhibition (*p* < 0.0001, Table 3, Figure 3). Three weeks later lake water from the same near-shore location (sample B2) and 200 feet from shore (sample B3) were submitted for analysis: MC-LA, MC-LR, MC-LF, and MC-RR were detected near shore and MC-LR, MC-LF, and MC-RR were detected 200 feet from shore. B2 and B3 had significant inhibition of PP1 activity (*p* < 0.0001, Table 3, Figure 3). The following week a third near-shore water sample was submitted (sample B4), and had low detectable levels of MC-LR, MC-LF, and MC-RR and no significant PP1 inhibition (*p* = 0.0727 Table 3, Figure 3). Samples from the dog were not available for testing.

The PPIA results of Clear Lake water samples confirm the presence of toxins capable of inhibiting PP1 one week after the dog’s sudden illness. In mammals, PP1 inhibition can result in extensive loss of hepatic architecture, hepatocyte necrosis and hepatic hemorrhage [43]. The dog’s rapid decline from nonspecific clinical signs to severe DIC in four days is likely the result of acute hepatic necrosis, which, combined with a history of swimming in a lake known to contain cyanobacteria [44] suggests MC toxicosis as the most likely cause of death. Furthermore, exposure to α-amanitin, a potent hepatotoxin in *Amanita* spp. mushrooms present in the Clear Lake area, was ruled out by a negative result of urine analysis. Leptospirosis, an important differential diagnosis due to the hemorrhagic presentation, was ruled out by post-mortem diagnostic testing.

The LRS of the PPIA model became less negative over the course of several weeks of sample collection, suggesting that the overall biological toxicity of the Clear Lake samples decreased over time. In agreement with the biological assay, the concentrations of each detected MC congener decreased over the month long sampling period. Interestingly, the congener composition also changed. MC-LA was only detected near shore in weeks one and four while MC-LR was detected in all collected water samples. MC-LF and MC-RR were detected in near and far shore samples in weeks four and five. MC-LR concentrations increased between weeks one and four, before decreasing to below week one concentrations in week five, suggesting a dynamic environment of cyanotoxin production and degradation during the month of August. The appearance of detectable concentrations of MC-LF and MC-RR and disappearance of MC-LA also support the idea of a dynamic and complex lake water environment, emphasizing the need to collect water samples quickly after a toxicosis event and regularly at various locations to detect potential health risks. The appearance of high concentrations of MC-LF was surprising and demonstrates the necessity to expand the list of traditional MC congeners (MC-LR, MC-RR, MC-YR and MC-LA) in monitoring efforts. MC-LF is considered hydrophobic [45] and has the potential to bind sediment, which, if disturbed, may result in MC release even without a concurrent bloom and further complicate diagnostic investigations and public health monitoring efforts.

With highly variable MC congener concentrations detected over the four-week period, the PPIA is a useful tool to detect PP1 inhibitors in dynamic cyanobacterial ecosystems. Chemical detection may underestimate MC concentrations and misjudge the risk of cyanotoxins. The first sample (B1) had the most potent PP1 inhibition, but did not contain the highest detected concentrations of MCs. Additional MC congeners not detectable by the LC-MS/MS method may have been present. Sample matrix composition appears to influence PPIA results with the potential to increase PP1 activity. Sample B4 had low detectable concentrations of MCs by LC-MS/MS but a positive PPIA linear slope. This finding suggests that the water contained compounds that were capable of catalyzing the formation of *p*NP by PP1, which was also observed in the sample A1 from the Kentucky case. It is possible that many water samples contain PP1 catalysts capable of the formation of *p*NP. Only water samples with sufficient MC concentrations to inhibit PP1 activity greater than the catalyst’s ability to produce *p*NP will result in significant negative linear slopes. This limitation is important to consider in the future use of the PPIA with environmental samples. Minimal environmental sample processing used in this study may play a role; therefore, additional cleanup procedures may reduce this problem but will require more time and costs. Our case investigation showed that the use of the PPIA with the linear regression model is suitable for identifying potent toxins that may escape detection by LC-MS/MS.

MCs can persist in natural waters in the dark for months or years, yet can undergo slow photochemical breakdown and isomerization in full sunlight, with over 90% breakdown in two–six weeks, depending on the presence of pigments and humic substances [46]. In this study, PP1 inhibitory activities decreased over the span of one month while individual MC congener concentrations increased and decreased over time. A combination of removal pathways, such as UV and bacterial degradation, dilution and dispersion throughout the lake, and/or bioaccumulation in fish and other organic matter may have played a role in the decreasing concentrations.

Samples from the dog were not available for testing to confirm the presence of cyanotoxins in the gastrointestinal tract or tissue samples. Historical data and regular water testing of Clear Lake have confirmed the presence of cyanotoxins for many years, and this provides a reasonable basis for our conclusion that the dog likely consumed a lethal dose of MCs. In particular, a few weeks before the dog’s death, concern over visible cyanobacterial blooms triggered the Lake County Department of Water Resources to collect water samples from three different locations for cyanobacteria identification by a phycologist (Green Water Laboratories, Palatka, FL, USA). Clear Lake Lower Arm, near the area of the dog’s lake access, contained *Woronichinia naegeliana* while Clear Lake Park contained *Microcystis panniformis*, *Woronichinia naegeliana*, *Microcystis wesenbergii* and *Microcystis botrys* and Clear Lake Upper Arm contained *Aphanizomenon cf flos-aquae*, *Gloeotrichia echinulata*, *Anabaena* sp. and *Woronichinia naegeliana*. These cyanobacteria were identified in Clear Lake previously during the summer months [44] and *Microcystis* spp., *Anabaena* spp. and *Gloeotrichia* spp. are all producers of MCs. Additionally, Clear Lake data from 2011 show highest MC concentrations between August and October in all areas of the lake. Based on all information available, it is very plausible that the dog died from MC toxicosis. To the best of the authors’ knowledge, this is the first known canine death from consuming harmful cyanobacteria toxins from Clear Lake [47]; the last report of known canine illness from cyanobacteria consumption from Clear Lake is from the late 1920s [48].

Apart from Clear Lake, other water sources in California have resulted in animal deaths with most reports from Northern California. Three dogs died in Castro Valley, five dogs died in Big Lagoon in 2001, and four dog deaths occurred after swimming in the South Fork of the Eel River in 2002 and 2004 [49,50]. In the US, between the late 1920s and 2012, an estimated 368 dogs died or developed illness after exposure to cyanotoxins [51]. In recent years, the continued and increasing risk for cyanotoxin exposure is illustrated by six dogs dying in 2014 and three in 2015 after swimming in or drinking cyanobacterial bloom-contaminated water [52,53,54,55,56]. The frequency of cyanotoxin exposure in animals is likely underreported due to the indiscriminant behavior of animals and absent diagnostic investigations. Beyond the US, the hazard of cyanotoxins is a global concern since the first report of cyanobacterial toxicosis of animals in 1878 from Australia [57], and with massive cyanotoxin blooms continuously reported from all over the world, including Lake Taihu, China [58] and Lake Erie, Ohio [59]. The PPIA with a linear regression model presented here ensures that the effects of potentially toxic PP inhibitors are accounted for and considered in veterinary diagnostic investigations. Valuable other assays to consider in investigations include ELISA, HPLC, and metagenomics; methodology details are beyond the scope of this publication. Most of these methods require a higher level of technical skill and advanced resources compared to the PPIA used here. The PPIA is a cost-effective and statistically sound method that should be used in parallel with LC-MS/MS analysis during cyanotoxins exposure investigations.

## 3. Experimental Section

### 3.1. Chemicals and Reagents

MC-LR was purchased from Sigma-Aldrich (St. Louis, MO, USA, discontinued) and MC-LR, MC-RR, MC-YR, MC-LA and MC-LF were purchased from Enzo Life Sciences (Farmingdale, NY, USA, 1 mg, purity of ≥95% (HPLC)). Okadaic acid was purchased from LC Laboratories (Woburn, MA, USA, 100 µg, purity 98%) and tautomycin was purchased from AG Scientific, Inc. (San Diego, CA, USA, discontinued, 100 µg, purity 97%) and Wako Chemicals (Richmond, VA, USA, 100 µg purity ≥90%). All chemicals were purchased from Sigma-Aldrich (St. Louis, MO, USA) at analytical grade or higher. MC-LR, MC-LF, okadaic acid and tautomycin were dissolved in 1 mL of 2.5% methanol and stored at −20 °C at final stock concentrations of 1,000,000 µg/L (MC-LR), and 100,000 µg/L (MC-LF, okadaic acid, tautomycin). Recombinant PP1 was purchased from EMD Millipore (Billericia, MA, USA, 100 U, 2500 U/mL, α-isoform, rabbit muscle, recombinant, *E. coli*). Stock PP1 was solubilized in storage buffer according to manufacturer (200 mM sodium chloride, 50 mM HEPES, 2.5 mM dithiothreitol (DTT), 1 mM manganese chloride (MnCl_2_), 0.1 mM ethylene glycol tetraacetic acid (EGTA), 50% glycerol, 0.025% TWEEN-20 detergent, pH 7) to a final concentration of 250 U/mL; aliquots of 40 µL were stored at −80 °C.

### 3.2. Sample Collection and Preparation

Water samples from Clear Lake, California, and Nelson County, Kentucky, were collected in 1 L amber glass bottles, shipped overnight to the University of California, Davis, and stored at 4 °C until further processing. Rumen samples were collected during necropsy at the Veterinary Diagnostic Laboratory, University of Kentucky, Lexington, KY, USA. For sonication, 10 mL of homogeneous water sample or liquid from rumen contents were sonicated using the Microson™ XL 2000 Ultrasonic liquid processor (probe model CML = 4, Newton, CT, USA) at 50% output on ice for 10 min. Samples were then centrifuged for 10 min at 4500 RCF. Supernatant was removed, spun again if sample had high turbidity; aliquots of 1.5 mL were stored at −20 °C.

### 3.3. Liquid Chromatography Mass Spectrometry/Mass Spectrometry

An aliquot of 500 µL of homogenous samples was filtered through a 0.2 µm Nylon membrane (EMD Millipore, Billericia, MA, USA) by 10,000 *g* force for 10 min in centrifuge tubes. A 10 µL sample was injected on to a ACQUITY UPLC BEH C18 column (Milford, MA, USA) (130 Å pore size, 1.7 µm particle size, 2.1 mm × 50 mm) for chromatographic separation. LC-MS/MS analysis was performed on an EVOQ (Bruker, Fremont, CA, USA) system. The mobile phase was (A) water containing 0.1% formic acid (*v*/*v*) and (B) methanol containing 0.1% formic acid (*v*/*v*). The column was eluted with 150 µL/min mobile phase. The 10 minute elution began with 50% B, then gradually increased to 90% B over 6 min, held at 90% B for 2 min, then changed back to 50% B within 1 minute and balanced for the last 1 minute.

The mass spectrometer was set to heated positive electrospray ionization (HESI) mode with spray voltage 4.5 Kv, cone temperature 300 °C, cone gas flow 15 A.U. (arbitrary unit), heated probe temperature 350 °C, probe gas flow 30 au and nebulizer gas flow 30 A.U. The LC-MS/MS characteristics of each microcystin congener are listed in Table 4. The LODs were determined based on the signal-to-noise ratio of 3:1, while the LOQs were calculated from the signal-to-noise ratio of 10 times the noise level. For concentrations below the LOQ but above the LOD, we used estimated values provided by the LC-MS/MS analysis software, Bruker MSWS 8.0.1 (Bruker, Billerica, MA, USA). Values below the LOD were reported as not detected.

### 3.4. Protein Phosphatase Inhibition Assay

Enzyme solution buffer (52 mM Tris-HCl (pH 7.0), 0.12 mM EGTA, 1 mM DTT (DTT in 0.01 M sodium acetate, pH 5.2), 2 mM MnCl_2_, 0.5 mg/mL bovine serum albumin (BSA)) and reaction buffer (62.5 mM M Tris-HCl (pH 8.1), 0.2 mM MnCl_2_, 26 mM magnesium chloride, 0.5 mg/mL BSA, 2 mM DTT) were freshly prepared and kept on ice.

For the 96-well plate colorimetric assay, rows A and H and columns 1 and 12 were not used due to previously reported temperature differences in those wells [60]. All wells contained a final volume of 250 µL. Plate blanks were five wells per plate containing reaction buffer. To optimize the assay, 10 µL of MC-LR or MC-LF (0, 0.125, 0.25, 0.5, 1 µg/L), tautomycin (0, 1.6, 3.3, 6.5, 13 µg/L) or okadaic acid (0, 100, 250, 375, 500 µg/L) were added to wells in triplicate (dilutions in reaction buffer prepared fresh for each assay). Twenty microliters PP1 (working solution diluted in enzyme buffer, final well concentration 0.5 U/mL) and 220 µL *p*NPP (working solution made with reaction buffer, final well concentration 100 mM) were added to initiate the colorimetric reaction. Substrate blanks were prepared in duplicate, and contained *p*NPP (final well concentration 100 mM). The negative assay plate control, with no toxin, represented the baseline PP1 activity for that plate, which was compared to each toxin exposed PP1 activity rate to determine if exposure to MC-LR, MC-LF, okadaic acid or tautomycin changed PP1 activity.

To run prepared environmental samples, the same method was followed as above (250 µL total final volume and 20 µL PP1), except 0, 2.5, 5, 10 or 20 µL of sample was added to wells in triplicate (final well concentrations 0%, 1%, 2%, 4%, or 8% of prepared sample, diluted in reaction buffer as needed) and 210 µL *p*NPP (final well concentration 100 mM) was added to initiate the colorimetric reaction. Substrate blanks contained 0, 2.5, 5, 10 or 20 µL of sample and *p*NPP (final well concentration 100 mM) to correct for sample pigment coloration and *p*NPP hydrolysis by the sample itself. PP1 with and without MC-LR were included as positive and negative assay plate controls to make sure PP1 inhibition could occur successfully if MCs were present. The negative assay plate control, with no MC-LR or sample extract, also represented the baseline PP1 activity for that plate, which was compared to each exposed PP1 rate, to determine if exposure to a sample changed PP1 activity.

Well ocular density (OD) readings were measured using a spectrophotometer (Molecular Devices SpectraMax M3, Sunnyvale, CA, USA) and recorded (SoftMax Pro software version 5.4, Molecular Devices Corporation, Sunnyvale, CA, USA) at 405 nm and 37 °C, every two minutes for 1.5 h. The rate of PP1 activity was measured as millimoles of *p*NPP hydrolyzed to *p*NP per nanogram PP1 per minute (mM *p*-NP produced/ng PP1/min). A linear rate was required for data interpretation; therefore the rate between 1000–5400 s was used for analysis. The rate between 0–1000 s for the substrate blank and samples were similar, while after 1000 s the substrate blank rate would plateau while the PP1 rate would continue to increase. Rate of hydrolysis was determined using Beer’s Law: C = A/(εl), where C = concentration of *p*NP, A = absorbance measured over time (OD/min), ε = 18,000 M^−1^·cm^−1^ (extinction coefficient for *p*NP) and L = 0.848 cm (pathlength for 250 µL for the 96-well plate, determined using potassium chromate at 405 nM, ε = 1355 M^−1^·cm^−1^). Plate blanks were subtracted from all *p*NPP hydrolysis rates. Average substrate blank hydrolysis rates were subtracted from the average enzymatic hydrolysis rates and then divided by the amount of PP1 used (1.563 ng). Each sample was tested in triplicate at least four times on different plates.

### 3.5. Statistical Analysis

Due to plate-to-plate variation, the exposed rate of PP1 activity was compared to baseline PP1 activity for that given plate, which had no toxin or sample included. Therefore, each data point presented is a ratio of the exposed PP1 rate over the baseline PP1 rate, *i.e.*, the change in PP1 activity when a given volume of toxin or sample is added to the reaction. The data were transformed by specifying the response variable as the relative activity change, measured as log (exposed PP1 activity rate/control PP1 activity rate). This allowed data to be analyzed with a linear regression to determine if there was a significant change in PP1 activity with increased toxin concentration or sample volume. Standard error of the data (*N* = 4 for all samples) is presented in the tables with the linear regression slopes. Data were grouped by toxin or sample and analyzed using a linear regression. Graphs represent the log (exposed PP1 activity rate/control PP1 activity rate) on the *y*-axis and the concentration of toxin or volume of prepared sample tested on the *x*-axis. The maximum likelihood model is represented on the graph as a black line with a 95% confidence interval (R program, ggplot2 [61]). Significance was attributed to *p* < 0.05.

## 4. Conclusions

Biological toxicity assays and quantitative MC analyses play critical roles in the diagnostic investigations of suspect cyanotoxin exposures. The PPIA with a linear regression analysis can effectively detect naturally occurring PP1 inhibitors. The method can differentiate between purified MCs, okadaic acid and tautomycin in agreement with previously published inhibitory constants. The functional toxicity assay serves an important purpose when evaluating samples for PP1 inhibitors that escape analytical detection. This approach ensures that the effects of potentially toxic yet non-tested MC congeners are accounted for and considered in veterinary diagnostic investigations. Both veterinary cases demonstrate that with minimal sample preparation it is possible to have potentially catalytic compounds, which in one case resulted in false negative PPIA data for MCs. Despite this limitation, our case investigation was greatly enhanced with the use of the PPIA. It is expected that the use of functional assays will play an increasingly important role in the diagnostic investigation of PP inhibitors in biological and water specimens.

## Figures and Tables

**Table 1:** Protein phosphatase 1 (PP1) activity after exposure to methanol (sample preparation control), microcystin-LR (MC-LR), microcystin-LF (MC-LF), tautomycin and okadaic acid. Each sample (*N* = 4), ran in triplicate with a substrate blank, is presented with standard error (SE). The trend in PP1 activity inhibition by each toxin, as determined by the magnitude of the linear regression slope (LRS) of the PPIA, is in agreement with previously published PP1 inhibitory constants (K*_i_*) [29]: MC-LR and MC-LF have similar PP1 inhibition potencies with the steepest negative significant LRS, followed by tautomycin and then okadaic acid.

Toxin	LRS	SE	*p* Value	PP1 K_i_ (µg/L)
Methanol	−0.359	1.29	0.785	-
MC-LR	−0.463	0.0423	<0.0001	0.04
MC-LF	−0.550	0.0402	<0.0001	-
Tautomycin	−0.0542	0.00473	<0.0001	0.33
Okadaic Acid	−0.000919	0.0000582	<0.0001	118

**Table 2:** Liquid chromatography-mass spectrometry (LC-MS/MS) and protein phosphatase inhibition assay (PPIA) results of water samples A1 and A2 and rumen content samples A3–A5 collected from a farm in Nelson County, Kentucky. Control rumen sample A6 was collected from a ruminating calf without exposure to cyanotoxins. Results from the PPIA assay are described as a linear regression slope (LRS), with corresponding standard error (SE) and *p* value. N.D. = not detected.

			PPIA		
	Sample Type	LC-MS/MS (µg/L)	LRS	SE	*p* Value
A1	Pond Water	N.D.	0.00419	0.000497	<0.0001
A2	Pond Water	LR 5.7	−0.0364	0.00536	<0.0001
A3	Rumen Contents	LR 1.3	−0.0211	0.00169	<0.0001
A4	Rumen Contents	LR 4.3	−0.0867	0.00420	<0.0001
A5	Rumen Contents	LR 1	−0.0166	0.00116	<0.0001
A6	Rumen Contents, Control	N.D.	−0.000120	0.000629	0.851

**Table 3:** Liquid chromatography-mass spectrometry (LC-MS/MS) and protein phosphatase inhibition assay (PPIA) results of water samples collected from Clear Lake, California. Sample collection began one week after a dog became fatally ill after exposure to lake water. Water samples were collected from near shore, where the dog swam, and 200 feet from shore. Results from the PPIA assay are described as a linear regression slope (LRS), with corresponding standard error (SE) and *p* value.

	Sample Collection			PPIA		
	Location	Week	LC-MS/MS (µg/L)	LRS	SE	*p* Value
B1	Near Shore	1	LA: 2.1	−0.0223	0.00216	<0.0001
			LR: 0.4			
B2	Near Shore	4	LA: 1.7	−0.0173	0.00201	<0.0001
			LR: 0.6			
			LF: 50			
			RR: 0.5			
B3	200 ft from shore	4	LR: 0.6	−0.0147	0.00168	<0.0001
			LF: 11			
			RR: 0.2			
B4	Near Shore	5	LR: 0.3	0.00188	0.000971	0.0727
			LF: 2.6			
			RR: 0.20			

**Table 4:** Liquid chromatography-mass spectrometry characteristics of microcystins (MC) isomers including the limit of detection (LOD) and limit of quantification (LOQ).

ID	Molecular Weight (Da)	LOD/LOQ (µg/L)	Retention Time (min)	Precursor Ion	Product Ion	Collision Energy
MC-RR	1038.2	0.2/0.6	3.19	519.7	135.0	34
MC-YR	1045.2	0.3/0.7	3.95	523.6	135.6	30
				523.6	507.8	30
				523.6	456.7	30
MC-LR	995.2	0.2/0.5	4.30	498.4	135.1	20
				498.4	430.5	20
				498.4	482.1	10
MC-LA	910.0	1.3/4.2	6.70	910.0	372.8	30
				910.0	399.5	30
				455.5	172.4	60
MC-LF	986.2	1.9/6.4	7.63	1008.0	510.5	74
				1008.0	504.2	73
